# Recent Advances in Antibody Therapy for Alzheimer’s Disease: Focus on Bispecific Antibodies

**DOI:** 10.3390/ijms26136271

**Published:** 2025-06-28

**Authors:** Han-Mo Yang

**Affiliations:** Division of Cardiology, Department of Internal Medicine, Seoul National University Hospital, Seoul 03080, Republic of Korea; hanname@gmail.com

**Keywords:** Alzheimer’s disease, antibody therapy, bispecific antibodies, amyloid-beta, tau, neuroinflammation, blood-brain barrier, monoclonal antibodies

## Abstract

Alzheimer’s disease (AD) impacts more than half a million people worldwide, with no cure available. The regulatory approval of three anti-amyloid monoclonal antibodies (mAbs), including aducanumab, lecanemab, and donanemab, has established immunotherapy as a therapeutic approach to modify disease progression. Its multifactorial pathology, which involves amyloid-β (Aβ) plaques, tau neurofibrillary tangles, neuroinflammation, and cerebrovascular dysfunction, limits the efficacy of single-target therapies. The restricted blood–brain barrier (BBB) penetration and amyloid-related imaging abnormalities (ARIA), together with small treatment effects, demonstrate the necessity for advanced biologic therapies. Protein engineering advancements have created bispecific antibodies that bind to pathological proteins (e.g., Aβ, tau) and BBB shuttle receptors to boost brain delivery and dual therapeutic effects. This review combines existing information about antibody-based therapy in AD by focusing on bispecific antibody formats and their preclinical and clinical development, as well as biomarker-based patient selection and upcoming combination strategies. The combination of rationally designed bispecific antibodies with fluid and imaging biomarkers could show potential for overcoming existing therapeutic challenges and delivering significant clinical advantages.

## 1. Introduction

The progressive neurodegenerative disease Alzheimer’s disease (AD) impacts about 50 million people worldwide through its effects on memory loss and cognitive deterioration and behavioral problems [1]. The economic burden from AD exceeds $1.3 trillion each year, and experts predict 153 million cases by 2050 [2,3]. AD’s multifactorial pathology involves extracellular amyloid-β (Aβ) plaques, intracellular tau neurofibrillary tangles (NFTs), chronic neuroinflammation, and cerebrovascular dysfunction, leading to synaptic loss and neuronal death [4,5]. The amyloid cascade hypothesis demonstrates that Aβ deposition creates a sequence of events that leads to tau pathology development, as well as microglial activation and cytokine release of IL-1β and TNF-α, resulting in neurodegeneration [6,7]. Neuroinflammation, driven by microglial and astrocytic activation, amplifies pathology, while cerebrovascular dysfunction impairs clearance of toxic proteins [8]. Synaptic loss in the hippocampus correlates strongly with cognitive decline [8]. Early diagnosis becomes possible through plasma p-tau217 biomarkers, which demonstrate greater than 90% accuracy; yet, these tools are inaccessible to most low- and middle-income countries [9,10].

Early attempts to treat AD with β-secretase inhibitors, together with Aβ vaccines such as AN1792, ended in failure due to toxic side effects and poor drug efficacy [11,12]. Monoclonal antibodies established a breakthrough when aducanumab (2021), lecanemab (2023), and donanemab (2024) obtained Food and Drug Administration (FDA) approvals [13,14,15]. Research shows that these anti-Aβ monoclonal antibodies (mAbs) decrease plaques by 60–80% (amyloid positron emission tomography [PET]) while slowing down cognitive decline by 20–35% for patients with early AD who have mini-mental state examination (MMSE) scores between 20 and 30 [16]. The therapeutic application of these antibodies faces three main obstacles, which include amyloid-related imaging abnormality (ARIA) complications in 20–40% of patients, poor blood–brain barrier penetration, and expensive pricing, at $26,500–$32,000 annually [17,18]. Early mAbs such as bapineuzumab failed because they either targeted nontoxic Aβ monomers or were administered too late, proving the necessity for multi-target strategies that begin at an early stage [19].

AD’s complex pathology, involving Aβ, tau, neuroinflammation, and vascular dysfunction, limits single-target mAbs, necessitating bispecific antibodies that simultaneously address multiple pathways [20]. Bispecific antibodies that combine targeting of two antigens, including Aβ/Triggering Receptor Expressed on Myeloid Cells 2 (TREM2) and Aβ/tau, serve to improve both delivery and effectiveness [20]. The designs include IgG–like formats or nanobodies that make use of knobs-into-holes engineering together with blood–brain barrier (BBB) transporters (e.g., Transferrin Receptor 1 [TfR1]) to enhance brain delivery by 8–10 times [21]. This review integrates advances in AD antibody therapies, emphasizing bispecific antibodies, their designs, preclinical/clinical development, and future directions, including trispecific antibodies and precision medicine.

## 2. Evolution of Antibody Therapies for Alzheimer’s Disease

The development of antibody therapies for AD progressed from initial failures to FDA approval of mAbs, which targets Aβ as well as tau and neuroinflammatory pathways. This section discusses the existing FDA-approved anti-Aβ mAbs while providing an overview of other relevant therapeutic strategies.

### 2.1. Anti-Aβ Monoclonal Antibodies

Anti-Aβ mAbs target the amyloid cascade, with three FDA-approved therapies as of May 2025: aducanumab (Aduhelm^®^), lecanemab (Leqembi^®^), and donanemab (Kisunla™) [22] (Table 1). These reduce plaques as well as cognitive decline but increase the risk of ARIAs [23].

**Aducanumab:** The human IgG1 mAb, called aducanumab by Biogen, targets Aβ aggregates that exist as plaques and oligomers [13]. The FDA provided accelerated approval to aducanumab in June 2021 based on the EMERGE trial (NCT02484547) results, which involved 1638 patients receiving 10 mg/kg of IV treatment every four weeks [24]. The medication resulted in a 60% reduction of amyloid PET plaques, together with a 22% slowdown of the Clinical Dementia Rating—Sum of Boxes (CDR-SB) decline in patients with mild cognitive impairment (MCI) or mild AD who had MMSE scores between 24 and 30 [24]. The ENGAGE trial (NCT02477800) demonstrated inconsistent effectiveness [25]. The occurrence of ARIA-Edema (ARIA-E) and ARIA-Hemorrhage (ARIA-H) was reported in 35% and 19% of patients, respectively, while Apolipoprotein E ε4 (APOE4) carriers exhibited higher frequencies [26]. NCT04241068 requires additional post-marketing trials for aducanumab [27]. The high price tag of $28,000 yearly and minimal therapeutic benefits reduce the treatment’s practical value [28].

**Lecanemab:** Eisai/Biogen developed lecanemab to specifically target Aβ protofibrils [14]. The FDA granted full approval to lecanemab in July 2023 following the CLARITY-AD trial (NCT03887455), which demonstrated that patients receiving 10 mg/kg biweekly had a 27% decrease in CDR-SB and 50% reduction in cerebrospinal fluid (CSF) protofibril counts among early AD patients (MMSE 22–30) [14]. Amyloid PET results showed a 60-centiloid decrease, and 68% of participants reached clearance during an 18-month period [29]. The ARIA-E and ARIA-H side effects resulted in three deaths among APOE4 homozygotes, although they affected 12.6% and 17.3% of patients, respectively [14]. The medication’s effectiveness, together with reduced ARIA events, make it preferable, but the need for biweekly infusions at $26,500 per year creates administration difficulties [30].

**Donanemab:** Eli Lilly developed donanemab to detect pyroglutamate-modified Aβ in plaques [15]. The FDA approved donanemab for full approval in July 2024 after the TRAILBLAZER-ALZ 2 trial (NCT04437511) showed it reduced integrated Alzheimer’s Disease Rating Scale (iADRS) by 35% in patients with early AD (MMSE 20–28) with low/medium tau levels during 18 months with 80% plaque clearance at 10 mg/kg IV every four weeks [15]. The study found that MCI participants who received the treatment experienced a 60% decline in their iADRS scores [31]. ARIA-E (24%, 6% symptomatic) and ARIA-H (31%) were higher in APOE4 carriers [32]. The practice of interrupting dosing after plaque clearance helps reduce costs to about $32,000 per year; yet, it creates safety issues with microhemorrhages [33]. Researchers evaluate extended outcomes through their current NCT05026866 studies [34].

**Other mAbs**: Gantenerumab (Roche) targets Aβ fibrils, reducing plaque burden by 50% in APP/PS1 mice but failing Phase III trials (NCT01224106) due to insufficient cognitive benefits [35]. Crenezumab (Genentech) targets Aβ oligomers, showing modest preclinical effects in Tg2576 mice but no efficacy in Phase III (NCT02670083) [36]. Solanezumab, targeting Aβ monomers, was ineffective in Phase III (NCT01900665) [37]. These failures highlight the need for multi-target therapies.

### 2.2. Newly Reported Aβ-Targeting Monoclonal Antibodies

Beyond currently approved or late-stage Aβ-targeting monoclonal antibodies, several promising candidates are emerging from preclinical and early clinical development, offering diverse mechanisms by targeting various Aβ species and epitopes involved in its pathology.

**mAb 1F12**: This antibody targets specific forms of Aβ and has shown therapeutic potential in preclinical models [38].

**mAb 2C6**: Known for its distinct binding profile to Aβ species, mAb 2C6 is under investigation for its plaque-clearing capabilities [39].

**mAb OC64**: An early-stage antibody, OC64 has demonstrated activity against certain Aβ aggregates [40].

**mAb 158**: This antibody targets protofibrillar Aβ, similar to lecanemab, and has been a subject of interest in earlier research [41].

**SAR228810/SAR255952**: These are antibodies developed by Sanofi/Sarepta, with SAR228810 targeting aggregated Aβ. Their development status and specific therapeutic benefits are being evaluated [42].

**AAB-003**: An earlier generation anti-Aβ antibody, AAB-003, was part of early clinical investigations into Aβ immunotherapy [43].

These emerging candidates highlight the ongoing efforts to refine Aβ-targeting strategies, potentially leading to more effective and safer treatment options.

### 2.3. Emerging Antibody Targets: Tau and Neuroinflammation

Anti-tau mAbs target extracellular tau aggregates to block propagation, but intracellular tau remains challenging [44]. Semorinemab (Phase II, NCT03289143) and gosuranemab (Phase II, NCT03352557) failed to reduce NFTs or slow cognitive decline in early AD, as intracellular tau limits antibody access [45]. Preclinical P301S mouse models showed reduced NFTs, but human translation has been unsuccessful [46].

Immunomodulatory antibodies enhance Aβ clearance via microglial pathways [47]. AL002, targeting Triggering Receptor Expressed on Myeloid Cells 2 (TREM2), reduced amyloid in 5xFAD mice, with Phase II trials (NCT04592874) ongoing [20,48]. NLR family pyrin domain containing 3 (NLRP3) antibodies decreased inflammation in Tg2576 mice, suggesting potential for neuroinflammatory modulation [49]. These approaches, while promising, risk excessive immune activation, necessitating careful engineering [50].

### 2.4. Lessons from Early Failures

Early mAbs (e.g., bapineuzumab, solanezumab) and vaccines (e.g., AN1792) failed due to targeting nontoxic Aβ monomers, late intervention, or toxicity [12,19,37]. Bapineuzumab’s Phase III trials (NCT00575055) showed no cognitive benefits, as it targeted monomers rather than toxic oligomers or fibrils [19]. AN1792 caused meningoencephalitis in 6% of patients, halting trials [12]. These failures underscored three key limitations of monospecific mAbs: (1) inability to address AD’s multifactorial pathology (Aβ, tau, neuroinflammation); (2) poor BBB penetration (0.1–0.3% of dose); and (3) high ARIA risks in APOE4 carriers [17,18]. Biomarker-driven trials, using CSF Aβ42 and tau PET, improved outcomes by enabling earlier intervention and patient stratification [51]. These lessons have driven the development of bispecific antibodies, which target multiple pathways (e.g., Aβ/tau, Aβ/TREM2) and enhance BBB delivery, offering synergistic therapeutic effects, as demonstrated in preclinical models [20,21].

## 3. Bispecific Antibodies: A Paradigm Shift in AD Therapy

Bispecific antibodies, engineered to bind two antigens, target AD’s multifactorial pathology, including Aβ, tau, and neuroinflammation [21]. As of May 2025, none are marketed for AD, but preclinical and early clinical data show significant promise [21,52]. The following sections demonstrate an in-depth analysis of their design, mechanisms, preclinical advances, clinical trials, BBB penetration strategies, and future directions.

### 3.1. Design and Engineering of Bispecific Antibodies

**Figure 1:**
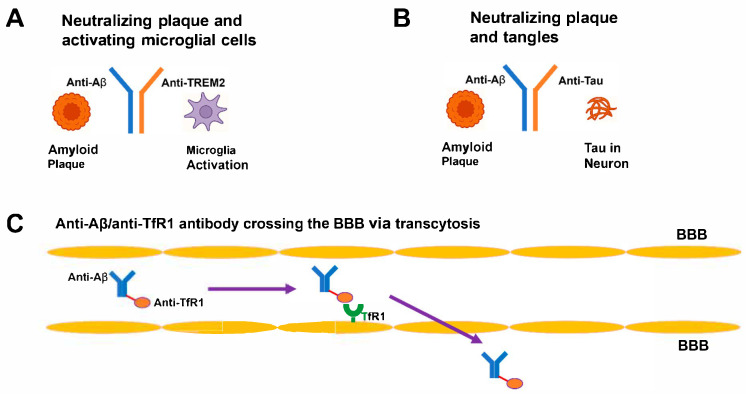
Mechanism of bispecific antibody in AD. (**A**) IgG–like antibody with arms targeting Aβ (blue) and TREM2 (red), binding plaques and activating microglia. (**B**) Anti-Aβ/anti-tau antibody neutralizing plaques and tangles. (**C**) Anti-Aβ/anti-TfR1 antibody crossing the BBB via transcytosis. Aβ, amyloid-beta; TREM2, Triggering Receptor Expressed on Myeloid Cells 2; TfR1, Transferrin Receptor 1; BBB, blood–brain barrier.

The construction of bispecific antibodies allows them to bind to two different antigens, which provides a diverse therapeutic approach for treating AD [20]. The molecular formats used in bispecific antibodies include IgG–like molecules with dual-binding arms as well as tandem single-chain variable fragments (scFv) for compact size, bispecific T-cell engagers (BiTEs) for immune activation, dual-variable-domain immunoglobulins (DVD-Ig) for enhanced stability, and nanobodies derived from camelid antibodies for superior tissue [53]. These formats allow precise applications: nanobodies penetrate through dense brain tissue, while IgG–like structures provide extended half-lives [54]. The main targets in AD therapy include Aβ and tau for removing plaques and tangles, Aβ and immune receptors (e.g., TREM2, CD33) to activate microglia, Aβ and BBB transporters (e.g., TfR1, low-density lipoprotein receptor-related protein 1 [LRP1]) to deliver to the central nervous system (CNS), and novel combinations such as Aβ/Beta-site Amyloid Precursor Protein Cleaving Enzyme 1 (BACE1) or tau/Glycogen Synthase Kinase-3 Beta (GSK3β) to decrease Aβ formation or tau phosphorylation [52]. The development of bispecific antibodies requires solving the issues of chain mispairing and aggregation [55]. The Fc region modification through the knobs-into-holes technique enables heterodimerization by making correct heavy-chain pairing possible [56]. The CrossMab method exchanges light- and heavy-chain domains to stop incorrect assembly while achieving more than 95% purity [57]. The simplified light-chain design in manufacturing allows production of identical light chains across both antigen-binding sites to decrease manufacturing expenses [57]. The optimization of binding affinity and specificity in bispecific antibodies is achieved through computational tools, which include molecular dynamics simulations and machine learning predictions for epitopes with dissociation constants (Kd) at low nanomolar levels [58]. For example, the Kd value for TfR1 binding of a bispecific antibody directed against Aβ and TfR1 reached 2 nM while showing enhanced BBB penetration by 10-fold in Tg2576 mice [59]. Humanization and glycoengineering techniques minimize immunogenicity, which decreases the formation of anti-drug antibodies in non-human primates [60]. The proteolysis-targeting chimeras (AbTACs) utilize the ubiquitin–proteasome system to break down intracellular Aβ aggregates in neuronal cells and show clearance in 5xFAD cultures [61]. These advancements result in stable bispecific antibodies that can be manufactured at scale for therapeutic use [20]. Figure 1 demonstrates these design principles in the AD brain.

**Bispecific Antibody Platforms:** Platforms like CrossMab, Knobs-into-Holes, Genmab DuoBody, BiTE, WuXiBody, SMABody, YBODY, and FIT-Ig vary in design and application (Table 2). CrossMab ensures high purity but requires complex production, while Knobs-into-Holes is scalable but moderately immunogenic. BiTEs activate T-cells but have short half-lives, limiting CNS use. IgG–like platforms (CrossMab, Knobs-into-Holes) are preferred for AD due to stability and half-life. These advancements enable scalable, stable bispecific antibodies for AD therapy.

### 3.2. Mechanisms of Action

The combined action of bispecific antibodies enables simultaneous targeting of multiple AD pathways, which enables better outcomes than monospecific mAbs [21]. The combination of anti-Aβ/anti-tau antibodies leads to a decrease in aggregated pathology in P301S/APP mice according to thioflavin-S staining [62]. The dual specificity of these antibodies prevents Aβ from activating tau phosphorylation, thus leading to stabilized microtubules and improvement in hippocampal synaptic density [62]. The combination of anti-Aβ/anti-TREM2 antibodies increases microglial phagocytic activity, which results in the removal of Aβ plaques in APP/PS1 mice above the clearance achieved by anti-Aβ mAbs through upregulating TREM2 signaling and CD68 expression [63]. The binding kinetics demonstrate high-affinity interactions between the antibody and its targets [47]. Anti-Aβ/anti-TfR1 antibodies use receptor-mediated transcytosis to boost brain concentrations by 10-fold in cynomolgus monkeys, which results in a 30% reduction of CSF Aβ42 [59]. The fusion protein αAβ-Gas6 activates microglia and astrocytes, which leads to Aβ plaque clearance via Tyro3, Axl, and MerTK (TAM) receptor activation without inducing inflammatory responses [64]. The targets Aβ/BACE1 and tau/GSK3β present dual opportunities for therapeutic intervention as they simultaneously block Aβ production and tau phosphorylation in APP/PS1 and P301S mouse models [65]. These mechanisms, including plaque clearance, tau stabilization, immune modulation, and enhanced delivery, make bispecific antibodies a groundbreaking therapeutic option [52].

### 3.3. Preclinical Advances and Classification of Bispecific Antibodies

Bispecific antibodies outperform mAbs in AD mouse models and are classified into two categories: (a) Pathology-targeting bispecific antibodies, engaging AD-related targets (e.g., Aβ/tau, Aβ/TREM2) for synergistic effects; and (b) BBB-shuttling bispecific antibodies, combining CNS-active antibodies with brain-penetrating modules (e.g., TfR1, CD98hc) to enhance delivery [59,63].

**Pathology-targeting bispecific antibodies**: Anti-Aβ/anti-TREM2 antibodies increased microglial plaque uptake in 5xFAD mice, reducing amyloid PET signals and improving Morris water maze performance [59]. CSF neurogranin and p-tau181 levels were reduced [66]. Anti-Aβ/anti-tau antibodies decreased plaques and NFTs in 3xTg-AD mice (immunohistochemistry), improving CA1 neuron long-term potentiation [62]. Anti-Aβ/anti-APOE4 antibodies reduced synaptic damage in APP/PS1 mice, lowering plasma neurofilament light (NfL) [67]. Anti-Aβ/anti-CD33 and anti-Aβ/anti-NLRP3 antibodies enhanced Aβ uptake and reduced inflammation (TNF-α, IL-1β) in Tg2576 and 3xTg-AD mice [68,69].

**BBB-shuttling bispecific antibodies**: Anti-Aβ/anti-TfR1 antibodies achieved 10-fold brain uptake in Tg2576 mice, reducing CSF Aβ42 by 30% [59]. Anti-Aβ/anti-CD98hc antibodies, leveraging the large neutral amino acid transporter, increased brain delivery 8-fold in APP/PS1 mice, with lower off-target effects than TfR1 [70]. Anti-Aβ/anti-BACE1 antibodies reduced Aβ generation in APP/PS1 mice, lowering soluble Amyloid Precursor Protein β (sAPPβ) [71].

Pathology-targeting bispecific antibodies like anti-Aβ/anti-tau reduced pathology by 30% in 3xTg-AD mice, but immunogenicity risks require glycoengineering [62]. These findings highlight bispecific antibodies’ potential to address multiple AD facets, though challenges like scalability and immunogenicity require further optimization.

### 3.4. Preclinical Advances

Bispecific antibodies have shown greater efficacy than mAbs in multiple AD mouse models based on preclinical studies [72]. The 5xFAD mice received an anti-Aβ/anti-TREM2 antibody, which increased microglial plaque uptake and resulted in reduced amyloid PET signals and better Morris water maze performance [63]. The markers of synaptic loss neurogranin and tau pathology p-tau181 in CSF were reduced [66]. The anti-Aβ/anti-tau antibody delivered to 3xTg-AD mice led to a reduction in plaque counts and NFTs (immunohistochemistry) while improving CA1 neuron long-term potentiation [62]. The combination of Aβ-APOE4-neutralizing anti-Aβ/anti-APOE4 antibodies reduced synaptic damage in APP/PS1 mice while lowering neurofilament light (NfL) in plasma [67]. The Tg2576 mice treated with anti-Aβ/anti-CD33 antibodies showed Aβ uptake enhancement along with a decrease in pro-inflammatory TNF-α levels [68,73]. The combination of anti-Aβ/anti-NLRP3 antibodies prevented inflammasome activation, which resulted in a decrease in IL-1β and tau phosphorylation in 3xTg-AD mice [49]. The combination of anti-Aβ/anti-BACE1 antibodies reduced APP/PS1 mouse Aβ generation while lowering soluble Amyloid Precursor Protein β (sAPPβ) [69]. The P301S mice received anti-tau/anti-GSK3β antibodies, which reduced AT8 phosphorylated tau while showing improved rotarod test motor function [71]. P301S/APP mice that received anti-Aβ/anti-TREM2 together with anti-tau/anti-GSK3β showed a decrease in pathology, which demonstrates potential synergistic effects [52]. These research findings demonstrate how bispecific antibodies can tackle multiple aspects of Alzheimer’s disease [63].

### 3.5. Clinical Development

Bispecific antibodies for AD treatment are being tested in early-stage clinical trials (Phase I/II), which evaluate drug safety together with pharmacokinetics and biomarkers, such as amyloid PET, CSF Aβ42, plasma p-tau217, and glial fibrillary acidic protein (GFAP) [63]. The market does not offer bispecific antibodies for AD management as of May 2025, although multiple candidates show promise [34].

**AL002 (Anti-TREM2/Anti-Aβ**): The product AL002 from Alector operates through two mechanisms by using TREM2 to stimulate microglia while using Aβ to eliminate plaques [63]. The Phase II trial (NCT04592874, INVOKE-2, planned n = 265, MMSE 22–30, 50–85 years) did not show significant effects on amyloid PET or various CSF biomarkers (e.g., p-tau, neurogranin) with various doses (15, 40, or 60 mg/kg biweekly) over 96 weeks [48]. ARIA-E and ARIA-H were reported, with a higher incidence in APOE4 homozygotes [48]. No significant effects on clinical outcomes like CDR-SB were observed. The extension study (NCT05744401) was discontinued [74].

**Anti-Aβ/Anti-TfR1:** Trontinemab (RO7126209), an anti-Aβ/anti-TfR1 bispecific antibody, is currently in Phase II development (NCT04639050, Brainshuttle AD study) [75]. Preclinical studies demonstrated significantly enhanced brain uptake (e.g., 4–18 fold increase in brain exposure) compared to conventional antibodies [75]. Interim results from the Phase Ib/IIa study (NCT04639050) have shown significant amyloid plaque reduction and early and significant reductions in CSF and plasma biomarkers, including total tau, phosphorylated tau 181 (p-tau181), p-tau217, and neurogranin [75]. A Phase III clinical program for trontinemab is planned.

The production complexity represents a major challenge, while the potential release of cytokines from immune-targeting antibodies and the need for reliable biomarkers are additional obstacles [20]. Studies investigate mAbs or small molecules used in combination therapy (e.g., NCT04241068) [27]. APOE4 status operates as the main modifying factor that leads to better trial results when combined with plasma p-tau217 and GFAP stratification [9,67,76].

### 3.6. Overcoming the Blood–Brain Barrier

The BBB restricts antibody transport so that only 0.1–0.3% of administered doses reach the brain [18]. The penetration of antibodies into the CNS improves through bispecific antibodies, which bind to BBB transporters [59]. However, bispecific and trispecific antibodies, due to their size, face similar or greater challenges [60]. BBB-shuttling bispecific antibodies and alternative strategies enhance CNS delivery (Table 3).

**TfR1 and CD98hc**: Anti-Aβ/anti-TfR1 antibodies increased brain uptake 10-fold in Tg2576 mice via transcytosis (Kd = 2 nM), reducing CSF Aβ42 by 30% [59,77,78]. Anti-Aβ/anti-CD98hc antibodies achieved 8-fold uptake in APP/PS1 mice, leveraging high BBB expression and lower off-target effects [79,80]. Trontinemab (anti-Aβ/anti-TfR1) showed 12-fold uptake preclinically [59].

**Alternative Strategies**: RVG peptides, targeting nicotinic acetylcholine receptors, increased anti-Aβ antibody uptake 3-fold in 5xFAD mice [81,82]. Insulin-receptor-mediated transport achieved a 5-fold uptake in APP/PS1 mice [83]. Lipid nanoparticles encapsulating anti-Aβ/anti-TREM2 antibodies increased delivery 12-fold in Tg2576 mice, reducing CSF p-tau181 [84]. Silicon-based materials are under exploration [84]. Focused ultrasound with anti-Aβ/anti-tau antibodies transiently opened the BBB in 5xFAD mice, reducing plaques and restoring spatial memory [85]. Adeno-associated virus (AAV) vectors sustained antibody production in APP/PS1 mice for 6 months, decreasing plaques [52].

These strategies, particularly TfR1, CD98hc, and nanoparticles, are clinically viable but require optimization to balance efficacy and safety.

### 3.7. Emerging Technologies and Future Directions

Emerging technologies are revolutionizing bispecific antibodies for AD by enhancing efficacy, specificity, and delivery. These innovations address AD’s multifactorial pathology but face technical and regulatory challenges.

**Trispecific Antibodies**: Trispecific antibodies, targeting three antigens, offer synergistic effects. A trispecific antibody binding Aβ, tau, and TREM2 reduced pathology by 40% in P301S/APP mice, decreasing thioflavin-S-stained plaques and AT8-positive neurofibrillary tangles, while improving Morris water maze performance by 30% [86]. However, manufacturing complexity results in low yields (<50% purity) and high costs (~1.5–2 times bispecific antibodies), with immunogenicity risks requiring Fc optimization [86]. However, in terms of bispecific and trispecific antibodies, their larger size exacerbates delivery issues.

**CRISPR-Based Gene Editing**: CRISPR/Cas9, paired with anti-Aβ/anti-tau bispecific antibodies, corrected APP mutations in APP/PS1 mice, reducing Aβ production by 35% and CSF Aβ42 levels via AAV delivery for 6 months [87]. Silencing PSEN1 in 5xFAD mice decreased plaque formation [87]. Off-target editing (5–10% in neuronal cultures), high vector costs ($500,000–$1 M/patient), and regulatory hurdles limit translation [88]. Improved Cas9 specificity and standardized delivery are critical.

**AI-Driven Antibody Design**: AI optimizes antigen selection and binding affinity. ML algorithms predicted Aβ/TREM2 and Aβ/tau pairs with Kd of 1–3 nM, and AI-optimized anti-Aβ/anti-TfR1 antibodies achieved 12-fold brain uptake in Tg2576 mice, clearing 20% more plaques than controls [59]. However, AI requires large datasets (>10,000 structures), often incomplete for AD antigens, and validation across diverse populations (e.g., APOE4 variants) [58]. Integrating single-cell RNA sequencing could enhance precision.

**Advanced Delivery Systems:** Anti-Aβ/anti-CD98hc antibodies, leveraging the large neutral amino acid transporter, achieved 8-fold brain uptake in APP/PS1 mice, reducing hippocampal Aβ with fewer off-target effects than TfR1 [79,80]. Lipid nanoparticles encapsulating anti-Aβ/anti-TREM2 antibodies increased delivery 12-fold in Tg2576 mice, lowering CSF p-tau181 by 25% [84]. Stability issues and production costs ($100,000/kg) hinder scalability [84]. Focused ultrasound with anti-Aβ/anti-tau antibodies reduced plaques by 30% in 5xFAD mice, but this requires specialized equipment [85].

**Combination Therapies**: Future trials (e.g., NCT05744401) will combine bispecific antibodies with gene therapies, small-molecule inhibitors, and neuroregenerative agents. Plasma p-tau217 assays enable precision medicine by stratifying patients [22]. However, scalability, regulatory hurdles for gene therapies, and safety (e.g., ARIA, cytokine release) remain challenges [20].

Future directions include cost-effective trispecific designs, precise CRISPR base editing, multi-omics AI integration, and scalable nanoparticle platforms to enable personalized, effective AD therapies.

## 4. Challenges and Limitations

Safety issues, manufacturing problems, and limited accessibility create substantial barriers for using mAbs and bispecific antibody therapies to treat AD. The following section outlines these challenges to assist future development efforts.

### 4.1. Safety Concerns

Anti-Aβ mAbs cause ARIAs, which result in edema (ARIA-E) and hemorrhage (ARIA-H) that affect 20–40% of patients, with a higher risk observed in APOE4 carriers [17]. The ARIA-E rate was 35% among aducanumab patients, with 12% experiencing symptoms and 19% having ARIA-H; lecanemab patients experienced 12.6% ARIA-E and 17.3% ARIA-H, with occasional fatal hemorrhages [14,26]. The treatment donanemab produced 24% ARIA-E and 31% ARIA-H [31,32]. Bispecific antibodies, like AL002, showed 12% ARIA-E and 8% ARIA-H, potentially worsened by immune activation (e.g., TREM2) [89]. Mitigation includes affinity optimization, glycoengineering to reduce immunogenicity, dose titration, and MRI monitoring [60]. APOE4 screening minimizes risks but increases costs [17].

### 4.2. Manufacturing and Cost

The production costs for bispecific antibodies are higher than mAbs because of their complex engineering and specialized purification requirements. The cost estimates for these therapies are higher annually versus the yearly expense for traditional mAbs. The entry of biosimilars to the market becomes delayed because of patent protections. Insurance coverage remains limited due to high treatment expenses, which results in aducanumab access for only 20% of U.S. patients [28]. The manufacturing process requires innovative methods to achieve cost reductions during production scale-up.

### 4.3. Timing and Access

The importance of early intervention in AD treatment remains high; yet, biomarkers are unavailable to diagnose many cases [10]. The global availability of plasma p-tau217 and amyloid PET tests reach below 10% of clinics, while their combined cost remains high [10]. Patients from low- and middle-income countries represent a small percentage of therapy recipients because these regions lack proper diagnostic facilities [10]. The United States has seen mAb treatment access limited to eligible patients because of both high costs and infusion challenges [28,30,33]. Global health initiatives attempt to enhance diagnosis, but they operate under limited funding constraints [88].

## 5. Conclusions

FDA-approved mAbs—aducanumab, lecanemab, and donanemab—reduce Aβ plaques by 60–80% and slow cognitive decline by 20–35% but face limitations like ARIAs in 20–40% of patients, poor BBB penetration, and high costs [13,14,15,17,30,36,37]. Bispecific antibodies, not yet marketed as of May 2025, target multiple pathways (e.g., Aβ/tau, Aβ/TREM2), showing promising pathology reduction in preclinical models, though trials like AL002 (NCT04592874) did not meet primary endpoints for biomarker improvements [22,23,26,58,61,69,72,78]. Challenges include manufacturing complexity and early diagnosis barriers, with biomarkers like p-tau217 accessible. 

In conclusion, antibody-based immunotherapy has inaugurated a new chapter in AD treatment, but current benefits remain incremental. Bispecific engineering has the potential to transcend BBB limitations, engage multiple disease pathways, and enhance safety profiles. Ongoing clinical trials will clarify whether bispecific antibodies achieve superior efficacy with acceptable tolerability. Continued optimization of shuttle valency, affinity, and effector function, coupled with biomarker-guided patient selection, is poised to deliver the next wave of transformative therapies.

## Figures and Tables

**Table 1:** Marketed Anti-Aβ Monoclonal Antibodies for Alzheimer’s Disease.

Antibody	Target	Clinical Trial	Efficacy	Safety (ARIA)	Approval Status	Cost/Year
Aducanumab	Aβ aggregates	EMERGE (n = 1638)	60% plaque reduction, 22% CDR-SB slowing	ARIA-E: 35%, ARIA-H: 19%	FDA Accelerated (2021)	~$28,000
Lecanemab	Aβ protofibrils	CLARITY-AD (n = 1795)	60-centiloid reduction, 27% CDR-SB slowing	ARIA-E: 12.6%, ARIA-H: 17.3%	FDA Full (2023)	~$26,500
Donanemab	Pyroglutamate Aβ	TRAILBLAZER-ALZ 2 (n = 1736)	80% plaque clearance, 35% iADRS slowing	ARIA-E: 24%, ARIA-H: 31%	FDA Full (2024)	~$32,000
Gantenerumab	Aβ fibrils	GRADUATE (n = 1965)	50% plaque reduction (preclinical)	ARIA-E: 25%, ARIA-H: 15%	Failed Phase III	N/A
Crenezumab	Aβ oligomers	CREAD (n = 813)	Modest preclinical effects	ARIA-E: 10%, ARIA-H: 5%	Failed Phase III	N/A

References [13,14,15,22,23,24,25,26,27,28,29,30,31,32,33,34,35,36,37]. Aβ, amyloid-beta; CDR-SB, Clinical Dementia Rating—Sum of Boxes; iADRS: integrated Alzheimer’s Disease Rating Scale; ARIA-E, amyloid-related image abnormality-Edema; ARIA-H, ARIA-Hemorrhage; FDA, Food and Drug Administration. N/A: Data cannot be found.

**Table 2:** Comparison of Bispecific Antibody Platforms for AD Therapy.

Platform	Advantages	Disadvantages	Suitability for AD
CrossMab	High purity (>95%), stable	Complex production	High; long half-life for CNS delivery
Knobs-into-Holes	Scalable, versatile	Moderate immunogenicity	High; cost-effective for AD
Genmab DuoBody	High yield, natural IgG structure	Limited CNS data	Moderate; needs further validation
BiTE	Potent immune activation	Short half-life, poor CNS penetration	Low; unsuitable for chronic AD therapy
WuXiBody	Flexible design	Complex manufacturing, high cost	Moderate; scalability challenges
SMABody	Simplified production	Limited preclinical data	Moderate; emerging for AD
YBODY	High stability, dual targeting	Complex engineering	High; promising for multi-target AD
FIT-Ig	High affinity, scalable	Moderate immunogenicity	High; suitable for CNS applications

References: [20,53,54,55,56,57,58,59,60,61]. BiTEs, bispecific T-cell engagers; CNS, central nervous system.

**Table 3:** Strategies for Enhancing BBB Penetration of Bispecific Antibodies.

Strategy	Mechanism	Preclinical Outcome	Suitability for Bispecific Antibodies
TfR1-mediated transcytosis	Binds TfR1 on BBB endothelial cells	10-fold uptake, 30% CSF Aβ42 reduction (Tg2576)	High; scalable but off-target risks
CD98hc-mediated transcytosis	Binds CD98hc on BBB endothelial cells	8-fold uptake, reduced hippocampal Aβ (APP/PS1)	High; lower off-target effects
RVG peptide delivery	Targets nicotinic acetylcholine receptors	3-fold uptake in 5xFAD mice	Moderate; limited uptake efficiency
Insulin receptor transport	Binds insulin receptors on BBB	5-fold uptake in APP/PS1 mice	Moderate; complex receptor targeting
Lipid nanoparticles	Encapsulates antibodies for delivery	12-fold uptake, reduced p-tau181 (Tg2576)	High; scalable but stability concerns
Focused ultrasound	Transiently opens BBB	Reduced plaques, restored memory (5xFAD)	Promising; requires specialized equipment

References: [18,59,60,63,70,71,77,78,79,80,81,82,83,84,85]. TfR1, Transferrin Receptor 1; CSF, cerebrospinal fluid; BBB, blood–brain barrier; Aβ, amyloid-beta.

## Abbreviations

AD	Alzheimer’s disease
Aβ	amyloid-beta
NFTs	neurofibrillary tangles
mAbs	monoclonal antibodies
FDA	Food and Drug Administration
PET	positron emission tomography
MMSE	mini-mental state examination
ARIA	amyloid-related imaging abnormalities
TREM2	Triggering Receptor Expressed on Myeloid Cells 2
BBB	blood–brain barrier
TfR1	Transferrin Receptor 1
CDR-SB	Clinical Dementia Rating—Sum of Boxes
MCI	mild cognitive impairment
ARIA-E	ARIA-Edema
ARIA-H	ARIA-Hemorrhage
APOE4	Apolipoprotein E ε4
CSF	cerebrospinal fluid
iADRS	integrated Alzheimer’s Disease Rating Scale
NLRP3	NLR family pyrin domain containing 3
scFv	single-chain variable fragments
BiTEs	bispecific T-cell engagers
DVD-Ig	dual-variable-domain immunoglobulins
LRP1	low-density lipoprotein receptor-related protein 1
CNS	central nervous system
BACE1	Beta-site Amyloid Precursor Protein Cleaving Enzyme 1
GSK3β	Glycogen Synthase Kinase-3 Beta
Kd	dissociation constants
TAM	Tyro3, Axl, and MerTK
NfL	neurofilament light
sAPPβ	soluble Amyloid Precursor Protein β
GFAP	glial fibrillary acidic protein
AAV	Adeno-associated virus
